# Long-term follow-up and quality of life in patients receiving extracorporeal membrane oxygenation for pulmonary embolism and cardiogenic shock

**DOI:** 10.1186/s13613-021-00975-6

**Published:** 2021-12-24

**Authors:** Andrea Stadlbauer, Alois Philipp, Sebastian Blecha, Matthias Lubnow, Dirk Lunz, Jing Li, Armando Terrazas, Christof Schmid, Tobias J. Lange, Daniele Camboni

**Affiliations:** 1 Department of Cardiothoracic Surgery, University Medical Center Regensburg, Franz-Josef-Strauss-Allee 11, 93053 Regensburg, Germany; 2 Department of Anesthesiology, University Medical Center Regensburg, Franz-Josef-Strauss-Allee 11, 93053 Regensburg, Germany; 3 Department of Internal Medicine II, University Medical Center Regensburg, Franz-Josef-Strauss-Allee 11, 93053 Regensburg, Germany

**Keywords:** ECMO, Pulmonary embolism, Quality of life

## Abstract

**Background:**

Since 2019, European guidelines recommend considering extracorporeal life support as salvage strategy for the treatment of acute high-risk pulmonary embolism (PE) with circulatory collapse or cardiac arrest. However, data on long-term survival, quality of life (QoL) and cardiopulmonary function after extracorporeal membrane oxygenation (ECMO) are lacking.

**Methods:**

One hundred and nineteen patients with acute PE and severe cardiogenic shock or in need of mechanical resuscitation (CPR) received venoarterial or venovenous ECMO from 2007 to 2020. Long-term data were obtained from survivors by phone contact and personal interviews. Follow-up included a QoL analysis using the EQ-5D-5L questionnaire, echocardiography, pulmonary function testing and cardiopulmonary exercise testing.

**Results:**

The majority of patients (*n* = 80, 67%) were placed on ECMO during or after CPR with returned spontaneous circulation. Overall survival to hospital discharge was 45.4% (54/119). Nine patients died during follow-up. At a median follow-up of 54.5 months (25–73; 56 ± 38 months), 34 patients answered the QoL questionnaire. QoL differed largely and was slightly reduced compared to a German reference population (EQ5D5L index 0.7 ± 0.3 vs. 0.9 ± 0.04; *p*  < 0.01). 25 patients (73.5%) had no mobility limitations, 22 patients (65%) could handle their activities, while anxiety and depression were expressed by 10 patients (29.4%). Return-to-work status was 33.3% (average working hours: 36.2 ± 12.5 h/per week), 15 (45.4%) had retired from work early. 12 patients (35.3%) expressed limited exercise tolerance and dyspnea. 59% (20/34) received echocardiography and pulmonary function testing, 50% (17/34) cardiopulmonary exercise testing. No relevant impairment of right ventricular function and an only slightly reduced mean peak oxygen uptake (76.3% predicted) were noted.

**Conclusions:**

Survivors from severe intractable PE in cardiogenic shock or even under CPR with ECMO seem to recover well with acceptable QoL and only minor cardiopulmonary limitations in the long term. To underline these results, further research with larger study cohorts must be obtained.

## Background

Despite growing scientific insights and constantly evolving therapeutic strategies, pulmonary embolism (PE) still poses a threat to patients of all ages.

The incidence of PE in the European Union is estimated at 0.95/1000 population per year [1]. Depending on the severity of vessel obstruction and right heart strain, clinical manifestations range from mild symptoms to sudden cardiac death.

Acute high-risk PE, defined as PE with sustained hypotension (systolic blood pressure (SBP)  < 90 mmHg or pressure drop  > 40 mmHg for  ≥ 15 min), obstructive shock (SBP  < 90 mmHg or need for vasopressors, and end-organ hypoperfusion), or cardiac arrest, is associated with a mortality of 25% in patients with cardiogenic shock and up to 65% for those requiring cardiopulmonary resuscitation (CPR) [2, 3].

Therapeutic strategies include reperfusion via anticoagulation, thrombolysis and catheter-based or surgical thrombectomy [3]. Before ECMO was routinely established, patients with high-risk PE and severely deteriorated cardiac function received surgical embolectomy even during CPR with an acceptable outcome [4]. Though recent studies indicate better long-term outcome and lower PE-recurrence, surgery is merely used if thrombolysis fails or is contraindicated [5].

ESC guidelines recommend extracorporeal membrane oxygenation (ECMO) in critically ill, unstable PE patients as a bridge to other reperfusion techniques [3]. Though various studies on short-term outcome and mortality of PE treated with ECMO exist, little is known about the long-term outcome, quality of life (QoL), recovery of the right ventricle (RV) and cardiopulmonary function of these patients.

This study reports on short-term and long-term outcome of patients who received ECMO for acute, high-risk PE with emphasis on QoL and cardiopulmonary function.

## Methods

### Patients

We retrospectively analyzed our institutional ECMO database for all patients requiring ECMO from February 2007 to July 2020. Within these 13 years, 1876 ECMO runs have been entered into our database. Of these, 119 (6.3%) patients received ECMO for acute high-risk PE and were included in this study. Seventy-three patients received ECMO with suspected PE, which was later confirmed by CT.

ECMO was indicated in patients with suspected or confirmed high-risk PE under CPR, in cardiogenic shock due to RV failure (increasing dose of epinephrine  > 0.03 µg/kg/min and noradrenalin  > 0.05 µg/kg/min with a SBP  < 90 mmHg for  > 1 h), lactate levels above 3 mmol/l, in severe respiratory failure (Horowitz index (P/F ratio)  < 100 mmHg for  > 12 h) or if systemic thrombolysis or catheter-based thrombectomy failed and surgical thrombectomy was contraindicated.

The primary support type consisted of venoarterial (VA) ECMO. However, in patients with a predominant respiratory failure and high pCO_2_ levels and cardiogenic shock stabilized with high doses of vasopressors, venovenous (VV) ECMO was established. Our group already published a detailed description on what type of support to use [6].

ECMO was implemented by a team of trained doctors, perfusionists and nurses. All patients received standardized laboratory testing including troponin and brain natriuretic peptide, as well as echocardiography.

Our detailed ECMO management and equipment has been described previously [7, 8].

ECMO exclusion criteria consisted of known irreversible, severe brain damage, terminal malignancy, trauma with uncontrollable bleeding, unwitnessed circulatory arrest and an existing, credible declaration that the patient refuses to receive life-prolonging therapy. Age per se was no exclusion criterion.

Data were obtained via review of the institutional ECMO database and patients’ medical records, including the electronic medical chart (Metavision V6.9.0.23 2019, iMDsoft, Düsseldorf, Germany).

### Follow-up and QoL analysis

All patients willing to participate were invited for personal interviews.

Blood samples were evaluated for N-terminal pro-brain natriuretic peptide levels (NT-proBNP) to unveil signs of heart failure.

A trained echocardiographer assessed RV-function via transthoracic echocardiography (TTE), including right ventricular end-diastolic diameter (RVEDD), tricuspid annular plane systolic excursion (TAPSE) and systolic pulmonary artery pressure (sPAP).

Pulmonary function test (PFT) (SentrySuite V3 10.2, Vyaire, Höchberg, Germany) and cardiopulmonary exercise testing (CPET) (Vyntus CPX, CareFusion, Höchberg, Germany) were performed if they were physically capable. Forced expiratory volume in 1 s (FEV1) and vital capacity (VC) were collected; the FEV1/VC ratio was calculated.

The CPET protocol (bicycle ergometer in upright position) consisted of 3 min of rest, followed by 3 min of unloaded pedalling at a rate of 50–60 rotations/min. and a ramp protocol using work rates (WR) of 5–20 Watts/min. (according to the enquired fitness level) with a total exercise duration of 8–12 min. The following parameters were collected continuously over the whole study: heart rate, oxygen uptake (VO_2_), carbon dioxide output (VCO_2_), end-tidal pO_2_ and pCO_2_ (PET-pO_2_; PET-CO_2_), respiratory rate, tidal volume. A capillary blood gas analysis was taken from the earlobe at rest and immediately after peak exercise. Calculated parameters consisted of: O_2_-pulse (VO_2_/HR), aerobic capacity (∆VO_2_/∆WR), minute ventilation (VE), VE/VCO_2_ ratio (as slope and over time), breathing reserve (BR), respiratory exchange rate (RER), alveolar–arterial difference in partial pressures of oxygen (AaDO_2_), arterial-to-end-tidal difference in partial pressures of carbon dioxide [P(a-ET)CO_2_]. Peak oxygen consumption (Peak-VO_2_) is presented as ml × min^−1^ × kg^−1^ and % predicted according to Wasserman [9].

QoL analysis was obtained using the EuroQol-5D-5L questionnaire (EQ-5D-5L), consisting of 2 parts:

The first part included health-related questions evaluating mobility, self-care, usual activities, pain/discomfort, or anxiety/depression. Each dimension comprises five levels: no, slight, moderate, severe, or extreme problems. The five dimensions result in a 5-digit number, describing the patient’s health status (best imaginable health state: 1–1–1–1–1; worst imaginable health state: 5–5–5–5–5). It can be converted into an index value, ranging from maximally 1 to values lower than 0.

The second part consisted of the EuroQol visual analogue scale (EQ-VAS) where the patient rates his health on a visual analogue scale (range 0–100%). It represents a quantitative measure of health outcome reflecting the patient’s own judgement.

The EQ-5D-5L index values and EQ-VAS of our study population were compared to an age-matched German reference population.

Six more questions were added: return-to-work status, groin problems due to cannulation, hospitalizations, or out-patient treatment due to cardiac or pulmonary problems, recurrence of deep lower leg thrombosis (DVT) or PE and New York Heart Association classification (NYHA).

Approval for this study was obtained from our institutional ethics board (Case number: 20-1905-104).

Individual patient consent was waived as this study was based on anonymized data from routine care.

### Statistical analysis

Statistical analysis was performed with IBM SPSS Statistics 25 (IBM Corp., Armonk, NY). For data collection before import into SPSS, we used Excel for Windows (Microsoft Corp., Redmond, WA, USA).

Continuous data were presented as mean with standard deviation. Normal distribution was formally tested with the Shapiro–Wilk test.

Categorical data were presented as frequencies and percentages.

Comparison of continuous variables was performed using the Student’s *t *test and the Mann–Whitney *U *test.

Categorical variables were compared using the Chi-square test. A *p *value  <  0.05 was considered statistically significant.

To identify predictors of survival to discharge, several parameters were analyzed including their odds ratios (OR) in a univariate fashion using regression analysis.

Kaplan–Meier survival analysis was used to estimate the proportion of survivors and visualized in survival curves.

Missing data were deleted listwise.

## Results

### Study population

From February 2007 to July 2020, 119 patients underwent ECMO implantation for high-risk PE. The majority of patients were placed on VA ECMO (87 patients, 73.1%), about one-quarter on VV ECMO (32 patients, 26.9%). Mean age was 51 ± 15 years (min 16 years; max 79 years). Most patients were male (*n*  = 69.6%). Patient characteristics are displayed in Table 1.

**Table 1:** Demographic data

	All patients (*n* = 119)	Survivors (*n* = 54)	Non-survivors (*n* = 65)	*p* value
Age (years)	50.9 ± 14.8	49 ± 15	52.5 ± 14.5	0.196
Male	69 (57.9%)	30 (55.6%)	39 (60%)	0.625
BMI (kg/m^2^)	29 ± 14	30.3 ± 7.5	32.3 ± 10	0.246
Pre-lactate (mmol/l)	9.6 ± 6.9	6.9 ± 5.3	14.1 ± 7.2	**< 0.001**
Pre-norepinephrine (µg/kg/min)	0.5 ± 0.7	0.6 ± 0.5	0.5 ± 0.5	0.111
Pre-epinephrine (µg/kg/min)	0.8 ± 1.2	0.14 ± 0.3	0.2 ± 0.3	**0.04**
Pre-MAP (mmHg)	55 ± 17	61 ± 15	44 ± 16	**< 0.001**
Pre-pH	7.2 ± 0.17	7.2 ± 0.15	7.05 ± 0.2	**0.005**
Pre-prothrombin time (%)	60 ± 27	64 ± 24	51 ± 27	**0.007**
Pre-D-dimer (mg/l)	16 ± 3.8	21.3 ± 13.3	28.4 ± 12.9	0.269
Peak NSE-level (µg/l)	146 ± 171	53 ± 32	214 ± 208	**0.002**
Peak fHb (mg/l)	489 ± 554	278 ± 388	578 ± 642	**0.007**
Preimplant CPR	80 (67.2%)	31 (57.4%)	49 (75.4%)	**0.039**
Duration of CPR (min)	55.1 ± 32.7	46.3 ± 27.6	58.9 ± 34.3	**0.158**
Average ECMO support (days)	6.6 ± 8.2	7.6 ± 7.7	5.8 ± 8.6	**< 0.001**
ICU stay (days)	16.6 ± 18.4	25.2 ± 20.6	9.5 ± 12.5	**< 0.001**
Overall hospital stay (days)	20.2 ± 22.7	33 ± 25.5	9.5 ± 12.5	**< 0.001**

Statistically significant *p*-values are reported in bold

*BMI* body mass index; *MAP* mean arterial pressure; *NSE* neuron-specific enolase; *fHb* free plasma hemoglobin; *CPR* cardiopulmonary resuscitation; *ICU* intensive care unit

Of the 80 patients (67%) receiving CPR, 52 (65%) were placed on ECMO under CPR, the remaining 28 (35%) after returned spontaneous circulation but persisting shock and RV failure less than 12 h prior to ECMO.

Cannulation was performed at our center (*n*  = 64, 53.8%) or at the referring hospital (*n*  = 45, 37.8%). Ten (8.4%) patients were cannulated out of hospital.

Fifty patients (42%) received thrombolytic therapy, 17 patients (14.3%) interventional or surgical thrombectomy prior to/on ECMO. The remaining patients (*n*  = 52, 43.7%) were treated with therapeutic anticoagulation alone.

### Short-term outcome

Successful weaning was achieved in 61 patients (51.3%).

Another 7 patients (6%) died during further hospital stay, resulting in a survival-to-discharge rate of 45.4% (*n*  = 54). Leading cause of death was brain death following prolonged CPR.

Causes of death on ECMO are depicted in Table 2.

**Table 2:** Causes of death on ECMO

Death on ECMO	*n* = 58
Cerebral	30 (51.7%)
LCO	7 (12.1%)
MOF	10 (17.2%)
Sepsis	4 (6.9%)
Withdrawal of therapy	4 (6.9%)
Miscellaneous	3 (5.2%)

*LCO* low cardiac output; *MOF* multi-organ failure

Survival-to-discharge rates did not differ significantly for patients treated with thrombolysis, interventional/surgical thrombectomy or anticoagulation alone (*p*  = 0.76).

Survivors had a longer ventilation time (400.6 ± 343.8 h vs. 203.52 ± 311.3 h; p = 0.001) and ECMO duration (7.8 ± 7.7 days vs. 5.8 ± 8.6 days; p = 0.023) compared to non-survivors.

Survivors also had a longer ICU (25 ± 21 days vs. 9.5 ± 12.5; *p*  < 0.001) and overall hospital stay (33 ± 25.5 days vs. 9.5 ± 12.5 days; *p*  < 0.001).

Patients requiring CPR prior to ECMO had a significantly lower survival-to-discharge rate with 38.8% (*p*  = 0.038).

### Parameters predictive of survival

Parameters associated with higher survival-to-discharge rates were less acidotic pH-levels [OR 43.8 (increase/1 unit); 95% CI 3.2–595; *p*  = 0.005], higher mean arterial pressure [MAP; OR 1.07(increase /1 mmHg); 95% CI 1.04–1.1; *p*  < 0.001] and higher prothrombin time [OR 1.02 (increase/1 unit); 95% CI 1.0–1.04; *p*  = 0.007] prior to ECMO implantation.

CPR prior to ECMO (OR 2.72; 95% CI 1.04–4.96; *p*  = 0.039), elevated lactate levels [OR 1.13 (increase/1 mmol/l); 95% CI 0.98–1.31; *p*  < 0.001] and epinephrine dose [OR 1.89 (increase/1 µg/kg/min); 95% CI 0.867–4.14; *p*  = 0.04] before ECMO treatment were associated with lower survival-to-discharge rates, as was the need for hemodialysis during hospital stay (OR 2.19; 95% CI 1.04–4.63; *p*  = 0.039).

None of the treatment modalities (in detail: surgical thrombectomy, thrombolysis, VA or VV support) could be identified as predictive factor for survival to discharge (*p*  = 0.76).

### Long-term outcome

**Fig. 1:**
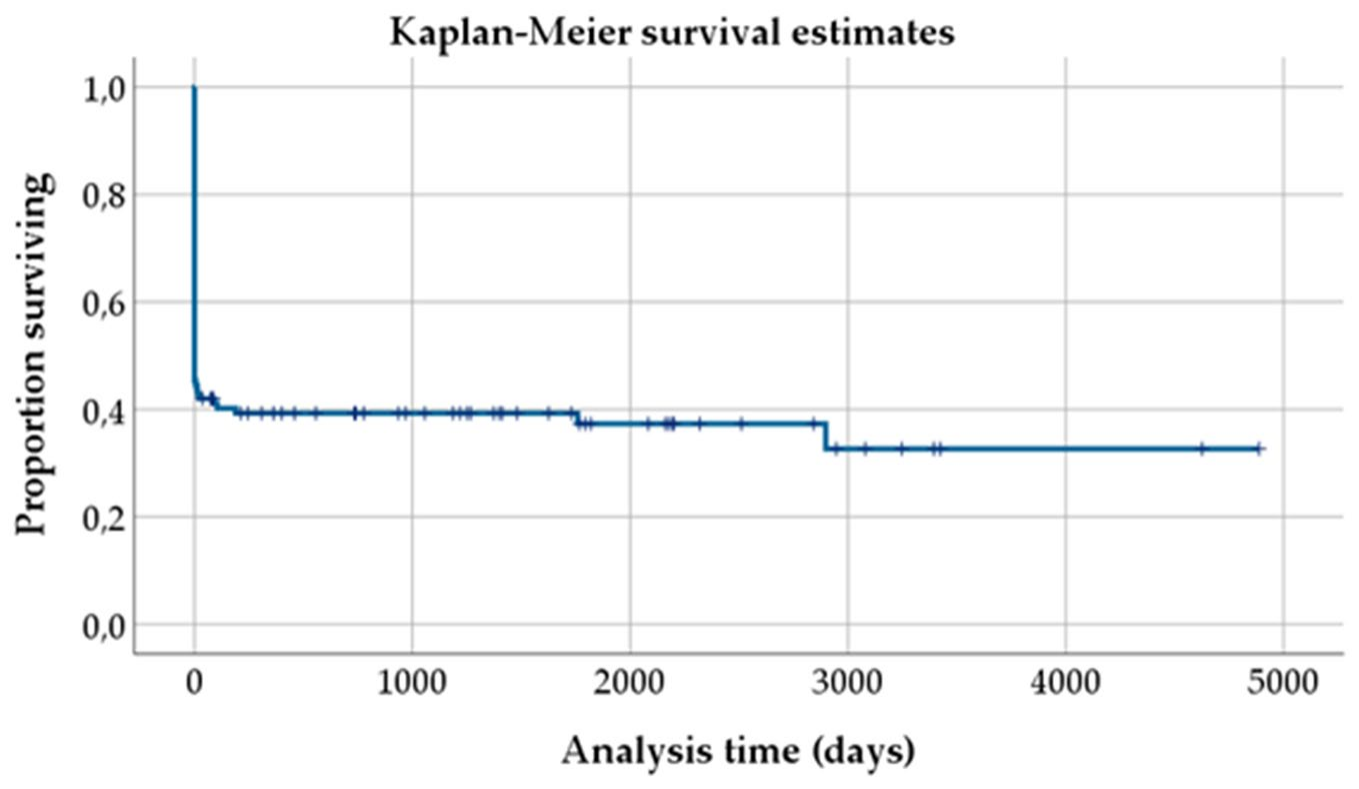
Kaplan–Meier estimation of survival over follow-up time. Figure 1 shows the overall survival (in days) after onset of pulmonary embolism until the time of follow-up

The 54 patients (45.4% of all patients) surviving to discharge were followed for 21.5 months (median 3 months); nine deceased during a mean follow-up time of 31.4 months (median 2.5 months; causes of death: 2 terminal malignancies, 3 recurrent PE, 4 unknown). 45 patients (37.8% of all patients) were available for follow-up. Kaplan–Meier survival curve is depicted in Fig. 1.

**Fig. 2:**
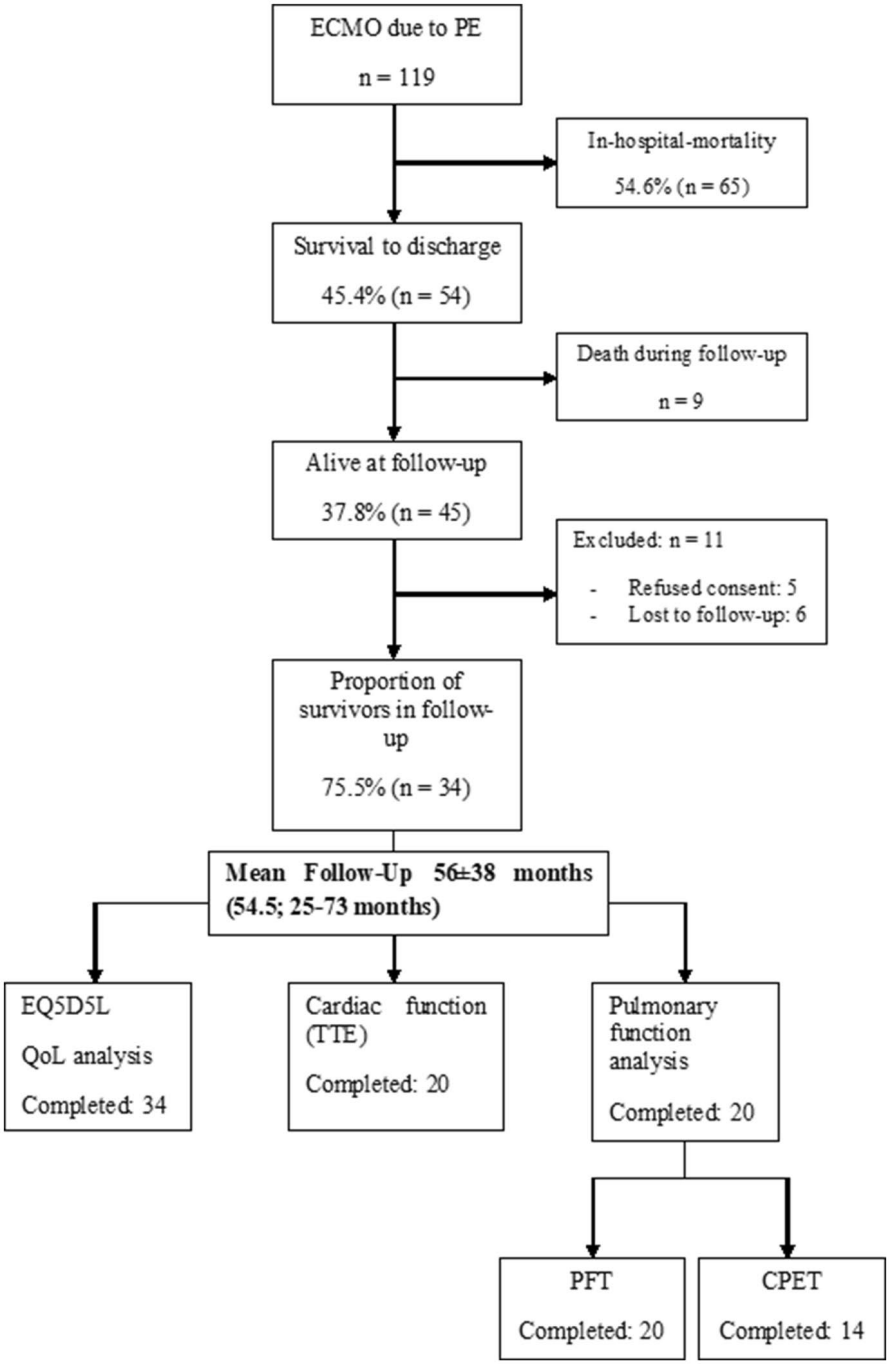
Flowchart of the recruitment and follow-up process. Figure 2 depicts the follow-up process from onset of the disease until follow-up

Six patients were lost during follow-up. Five patients refused to participate in this study. At a median follow-up of 54.5 months (25–73; 56 ± 38 months), 34 patients (75.5%; 34/45) patients answered the EQ-5D-5L, but only 51% (20/34) attended the follow-up examinations at our clinic. Follow-up is depicted in Fig. 2.

Of the interviewed patients, 11 (33.3%) had returned to work (average working hours: 36.2 ± 12.5 h/per week), 15 (45.4%) had retired from work early.

Ongoing symptoms of polyneuropathy were reported by three, recurrent DVT despite adequate anticoagulation with NOAC by two patients.

None of the patients reported groin problems after cannulation or experienced recurrent PE.

### Structured interviews and QoL

No limitations in motoractivity and mobility were stated in 73.5% (25 patients). Of 9 patients reporting moderate or severe problems, two had suffered irreversible spinal cord injury and were bound to a wheelchair; another 3 were hemiplegic due to cerebral insult. The remaining 2 patients suffered from joint pain. None of the limitations were due to PE or ECMO.

Twenty-two patients (65%) could handle their usual activities without or with only slight problems, 18 patients (53%) reported persistent pain and discomfort, mainly due to critical illness polyneuropathy.

Anxiety or depression was reported by 10 patients (29.4%).

**Fig. 3:**
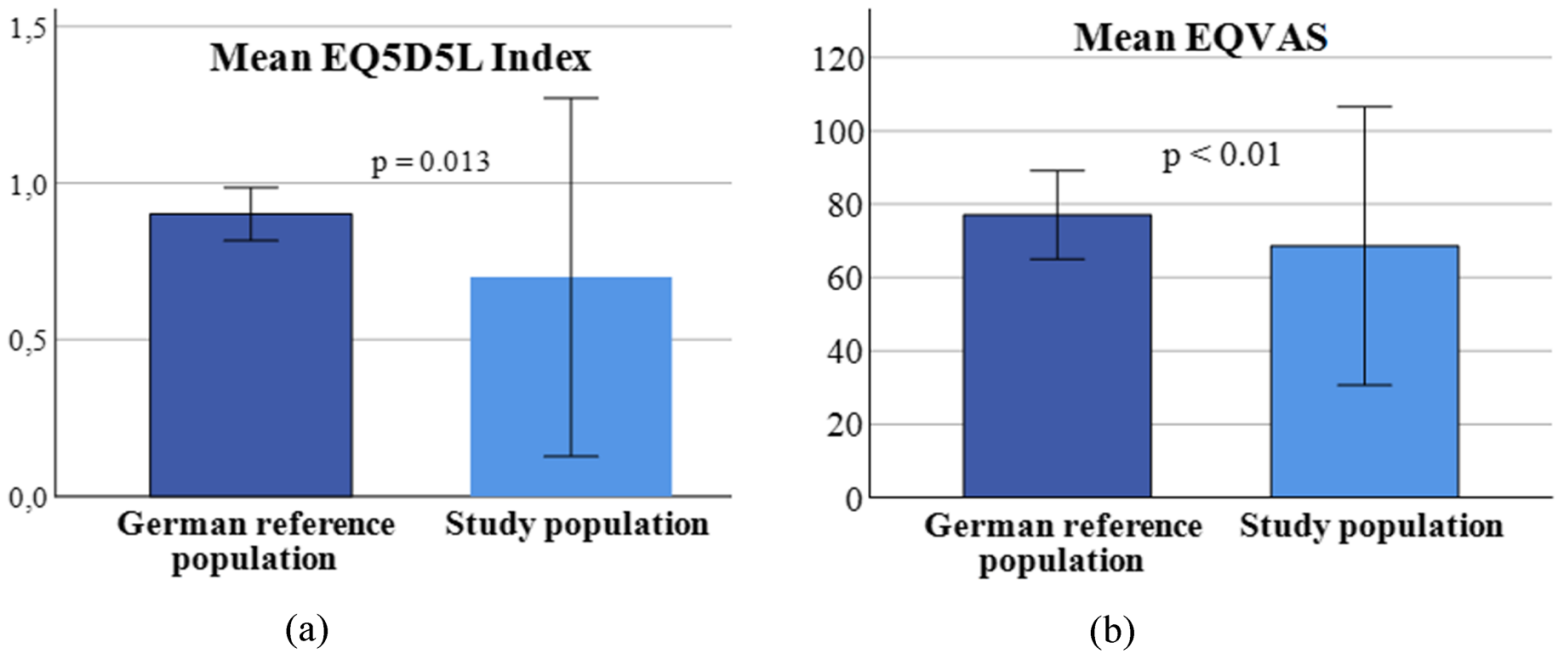
QoL comparison between study group and German reference population: **a** mean EQ5D5L index; **b** mean EQ-VAS. Figure 3 visualizes the differences in objective (EQ5D5L index) and subjective quality of life (EQ-VAS) of the study population and a German reference population

The mean EQ5D5L index of our study population was significantly reduced compared to an age-matched German reference population (EQ5D5L index study population = 0.74 ± 0.3; vs. German reference population = 0.9 ± 0.04; *p*  < 0.01). The EQ-VAS value also differed significantly between the groups (EQ-VAS study group = 64 ± 18%; vs. EQ-VAS German reference population  = 77.1 ± 6%; *p*  < 0.01) [10]. Results are visualized in Fig. 3.

The mean EQ5D5L index showed no difference between the 20 patients who attended the follow-up examination and the 14 patients who answered the questionnaire at home (EQ5D5L index exam group 0.7 ± 0.3 vs. at home group 0.79 ± 0.2; *p*  = 0.73), while the mean EQ-VAS showed a trend to better performance in the exam group (EQ-VAS exam group 68 ± 19% vs. 58 ± 15%; *p*  = 0.064).

Chest pain, dyspnea, vertigo or severe cardiopulmonary limitations were denied by 22 patients (64.7%), while 7 patients (20.5%) expressed limited exercise tolerance and dyspnea according NYHA Stadium II, 5 (14.7%) even NYHA III.

### Echocardiography, pulmonary function and cardiopulmonary exercise test

The entire group of 20 patients who attended our clinic for follow-up received TTE and PFT, while only 14 were willing and able to perform CPET. Of those, one patient was excluded from analysis due to ventilatory limitation caused by a pre-existing severe chronic obstructive pulmonary disease (FEV1 33% predicted, BR 2%, peak-VO_2_ 53% predicted). The results of the remaining patients are displayed in Table 3.

**Table 3:** Echocardiography, pulmonary function and cardiopulmonary exercise test at follow-up

Parameter (*n*)	Mean ± SD	Range (min.–max.)
Echocardiography (20 patients)		
Right atrial area, cm^2^	16.0 ± 3.6	8.0–22.0
RVEDD, mm	32.9 ± 5.6	22.0–44.0
TAPSE, mm	23.2 ± 5.8	17.0–38.0
Systolic PAP, mmHg (15 patients)	19.3 ± 4.8	13.0–30.0
Pulmonary function test and capillary blood gas analysis (20 patients)		
Vital capacity, % predicted	83.1 ± 16.1	51.0–105.0
FEV1, % predicted	80.5 ± 19.8	33.0–105.0
DLCO, % predicted	73.1 ± 22.1	30.0–97.0
pCO_2_, mmHg	36.0 ± 4.0	28.8–42.7
Oxygen saturation, % on room air	95.8 ± 1.5	94.0–98.0
Cardiopulmonary exercise test (13 patients)		
Maximum work rate, W	125.8 ± 52.8	52.0–221.0
Maximum work rate, % predicted	82.8 ± 23.1	41.0–118.0
Peak-VO_2_, ml × min^−1^ × kg^−1^	19.2 ± 8.3	9.3–34.0
Peak-VO_2_, % predicted	76.3 ± 16.1	44.0–102.0
RER max.	1.4 ± 0.2	1.1–1.9
Breathing reserve, %	44.4 ± 14.5	21.0–70.0
VE/VCO_2_ slope	28.7 ± 4.9	19.5–39.1
∆VO_2_/∆WR slope, ml × min^−1^ × W^−1^	9.6 ± 1.0	7.1–11.2
P(a-ET)CO_2_, mmHg	4.5 ± 2.0	0.3–6.9
AaDO_2_, mmHg	29.2 ± 7.0	19.9–40.1

*AaDO*_*2*_ alveolar–arterial difference in partial pressures of oxygen; *DLCO* diffusion capacity of the lung for carbon monoxide; *FEV1* forced end-expiratory volume in one second; *PAP* pulmonary arterial pressure; *P(a-ET)CO*_*2*_ arterial to end-tidal difference in partial pressures of CO_2_; *RER* respiratory exchange rate; *RVEDD* right ventricular end-diastolic diameter; *TAPSE* tricuspid anterior plane systolic excursion

There were no echocardiographic signs of relevant residual right heart strain or pulmonary hypertension, supported by NT-proBNP levels in the age-adjusted normal range for all patients. PFT revealed slightly reduced mean FVC, FEV1, and DLCO, reflecting mainly outliers with pre-existing lung diseases. None of the patients needed oxygen at rest. Hyperventilation at rest, which might reflect residual pulmonary vascular obstruction, was only evident in 2 patients below a pCO_2_ of 30 mmHg (28.8 and 29.4 mmHg). However, those patients had a Peak-VO_2_ of 75 and 95% predicted without further evidence for chronic thromboembolic pulmonary hypertension (CTEPH).

On CPET, the mean maximal WR and Peak-VO_2_ were slightly reduced. However, only 5 patients had a Peak-VO_2_ below 75% of predicted, and none of them showed a typical pattern of cardio-circulatory limitation. The good mean exercise performance of patients who were able to undergo a CPET study is further supported by normal mean values for the VE/VCO_2_ slope, aerobic capacity and gas exchange parameters (see Table 3).

## Discussion

To our knowledge, this is the largest single-center study analyzing the long-term outcome of patients requiring ECMO for acute high-risk PE, comprising 119 patients and covering 13 years of our institutional experience. 34 patients were evaluated for long-term outcome and QoL. Cardiopulmonary function and exercise capacity were examined in 20 patients.

For patients suffering from this severe disease with high morbidity and mortality, the hospital discharge rates in literature vary between 62, 70.1 and even 100% in patients receiving ECMO with anticoagulation treatment only [11, 12]. However, those reports investigated smaller cohorts (max. 21 patients) and included case reports which are prone to publication bias. More recent research with comparable study design was in line with our results, displaying survival-to-discharge rates of 44.4 and 38.5% [13, 14].

Our survival to discharge was 45.4%, overall survival at a mean follow-up of 4.5 years and a maximum of 13 years was 37.8%. It should be emphasized that most of our patients received ECMO during or after CPR.

Our findings are also supported by the most recent data of the international ELSO registry [6, 15].

CPR prior to ECMO was necessary in 67.2% of our patients and associated with an unfavorable prognosis, as reported by Corsi and Chen [13, 16].

CTEPH, defined as a mean pulmonary artery pressure  > 25 mmHg and a normal wedge pressure on right heart catheterization at rest due to residual pulmonary artery obstruction on chronic (> 3 months) therapeutic anticoagulation, is described with an incidence of 4% in PE survivors and decreases QoL and exercise capacity [17]. Even if we did not perform ventilation/perfusion scanning of the lung, given the findings of TTE, PFT, and CPET, it is unlikely that one of our patients who attended our center for follow-up suffered from CTEPH or relevant residual PE.

To our knowledge, no literature examining RV-function and cardiopulmonary function after PE treated with ECMO exists to date. Sista and Albaghdadi et al. performed research concerning these outcome variables in PE patients without ECMO treatment:

Sista reported a pooled prevalence of RV dysfunction on echocardiography of 18.1% after 6 months of follow-up, and a mean 6-min-walk distance of 415 m at 12 months [18]. Albaghdadi used echocardiography and CPET to evaluate cardiopulmonary performance and found RV dilation or dysfunction in more than one-third of PE survivors. Sixty percent showed a reduced peak-VO_2_ after 1 and 6 months [19]. However, this study did not include ECMO as treatment modality and patients were limited by deconditioning rather than residual PE or CTEPH.

To analyze our study population for signs of CTEPH, we assessed cardiopulmonary function and exercise capacity: after a mean follow-up of 53 months none of the patients had signs of RV dysfunction or pulmonary hypertension. All but 5 patients showed good exercise capacity with a Peak-VO_2_ of at least 75% predicted, including 8 (57%) patients who were resuscitated prior to ECMO.

In Albaghdadi’s and our study, there was no clear relationship between impaired exercise capacity and reduced RV-function or reduced pulmonary capacity. As data concerning exercise capacity prior to the event are lacking, it is impossible to refer to pre-existing conditions. However, exercise intolerance could be attributed to clearly pre-existing conditions like severe COPD. In other words, it was related rather to general deconditioning as in Albaghdadi’s experience.

Their data are however contradictory to our study regarding echocardiography on follow-up. No severe RV dysfunction or relevant pulmonary hypertension was noted in our patient population. Possible explanations for this discrepancy are a protective effect of ECMO on RV through workload reduction and better gas exchange, or different anticoagulation and medical treatment, even if selection bias is the most likely reason.

As a limitation of our study, only 20 of 34 eligible patients presented to our hospital for personal re-assessment, and only 14 were able and willing to perform a CPET study. Therefore, a considerable selection bias must be considered.

When evaluating new, invasive and expensive therapeutic strategies, not only survival, but also QoL has a high priority. Wenger et al. described QoL as ‘an individual’s perceptions of his or her functioning and well-being in different domains of life’ [20].

Only few studies concerning QoL after ECMO in general exist so far.

Corsi et al. interviewed 7 patients at a mean follow-up of 19 months post-hospital discharge using the SF-36 and found QoL impaired compared to a sex- and age-matched control [13]. Similar findings are reported by Sertic, with patients strongly limited concerning physical, but preserved mental health [21].

Hodgson’s group reported on QoL in ECMO for cardiogenic shock or respiratory failure, showing reduced QoL compared to the reference population with about 25% return-to-work status, similarly to our study (return-to-work status 34.7%) [22, 23].

A Norwegian study evaluated QoL of 213 patients after PE without ECMO treatment: the EQ-5D-5L index was 0.8 whereas EQ-VAS was 67% and was slightly reduced compared to an age-matched reference population [24]. As information about the underlying treatments or the number of patients receiving CPR in their study is lacking, a direct comparison to our results is highly difficult and no conclusions can be drawn concerning the impact of ECMO treatment on QoL of PE patients. However, the disease itself and prolonged ICU stay seem to be determining factors [25].

To assess the complex, multivariable and completely subjective entity of QoL, we chose the EQ-5D-5L questionnaire because it is a scientifically proven, simple, reliable test which includes physical, functional and psychological aspects.

According to EQ-5D-5L, QoL in our study population was reduced compared to a German reference population [26, 27]. The patients who answered the questionnaire at home showed significantly better index values while estimating their own QoL with EQ-VAS significantly worse than the examined patients. The reduced values of the self-estimated EQ-VAS of the patients at home might explain why these patients did not consent to personal interviews at our clinic. The ongoing Corona pandemic during the study period might also have contributed to a limited willingness to visit our clinic in person.

Therefore, our findings concerning QoL are consistent with previously published literature. To compare QoL in PE patients with and without ECMO treatment properly, further prospective trials are needed.

### Limitations

The study’s limitations lie in the retrospective design, reporting a single-center experience analyzing long-term functional follow-up after severe PE and ECMO with a relatively small study population. As it was our primary intention to evaluate the functional capacity of these patients, we did not divide the results according to VV and VA ECMO. The small sample size of VA and VV ECMO patients would have made proper comparison impossible. Still, this must be considered as limitation. Only 51% of patients received TTE and PFT or CPET at our clinic, therefore, a strong selection bias must be considered. Because of the small sample size, the results cannot be generalized and further studies with a larger follow-up group are much needed. As comparative QoL data are lacking before the event, it is uncertain if QoL was impaired before or because of ECMO treatment.

### Conclusions

ECMO should be considered in high-risk PE with cardiogenic shock or severe respiratory failure if other therapy modalities are not applicable. Survivors seem to recover well in the long term with an acceptable QoL and good overall cardiopulmonary function. Further research with larger patient cohorts is needed to underline these results.

## Data Availability

The datasets used and/or analyzed during the current study are available from the corresponding author on reasonable request.

## Abbreviations

AaDO_2_: Alveolar–arterial difference in partial pressures of oxygen
BR: Breathing reserve
COPD: Chronic obstructive pulmonary disease
CPET: Cardiopulmonary exercise testing
CPR: Cardiopulmonary resuscitation
CT-scan: Computed tomography scan
CTEPH: Chronic thromboembolic pulmonary hypertension
DLCO: Diffusion capacity of the lung for carbon monoxide
DVT: Deep vein thrombosis
ECMO: Extracorporeal membrane oxygenation
EQ-5D-5L: EuroQol-5D-5L questionnaire
EQ-VAS: EuroQol visual analogue scale
ESC: European Society of Cardiology
FEV_1_: Forced end-expiratory volume in one second
FVC: Forced vital capacity
ICU: Intensive care unit
LCO: Low cardiac output
LDH: Lactate-dehydrogenase
LV function: Left ventricular function
MAP: Mean arterial pressure
MOF: Multi-organ failure
NOAC: Novel oral anticoagulants
NSE: Neuron-specific enolase
NT-proBNP: N-terminal pro-brain natriuretic peptide
NYHA: New York Heart Association
OR: Odds ratio
P(a-ET)CO_2_: Arterial to end-tidal difference in partial pressures of CO_2_
PE: Pulmonary embolism
Peak-VO_2_: Peak oxygen consumption
Peak predicted-VO_2_: Predicted peak oxygen consumption
PET-pCO_2_: End-tidal carbon dioxide
PET-pO_2_: End-tidal oxygen
P/F ratio: Horowitz index
PFT: Pulmonary function testing
fHb: Free plasma hemoglobin
QoL: Quality of life
RER: Respiratory exchange rate
RV: Right ventricle
RVEDD: Right ventricular end-diastolic diameter
SBP: Systolic blood pressure
SF-36: Short Form-36 health survey
sPAP: Systolic pulmonary artery pressure
TAPSE: Tricuspid annular plane systolic excursion
TTE: Transthoracic echocardiography
VA: Venoarterial
VC: Vital capacity
VV: Veno-venous
